# Integrative *In Silico* Investigation Reveals the Host-Virus Interactions in Repurposed Drugs Against SARS-CoV-2

**DOI:** 10.3389/fbinf.2021.763540

**Published:** 2022-01-11

**Authors:** Wenhui Yu, Yuxin Bai, Arjun Raha, Zhi Su, Fei Geng

**Affiliations:** ^1^ Faculty of Health Sciences, McMaster University, Hamilton, ON, Canada; ^2^ Department of Mechanical Engineering, McMaster University, Hamilton, ON, Canada; ^3^ W Booth School of Engineering Practice and Technology, McMaster University, Hamilton, ON, Canada

**Keywords:** chloroquine, hydroxychloroquine, *in silico*, interactome mapping, SARS-CoV-2, toll-like receptor 9

## Abstract

The ongoing COVID-19 outbreak have posed a significant threat to public health worldwide. Recently Toll-like receptor (TLR) has been proposed to be the drug target of SARS-CoV-2 treatment, the specificity and efficacy of such treatments remain unknown. In the present study we performed the investigation of repurposed drugs via a framework comprising of Search Tool for Interacting Chemicals (STITCH), Kyoto Encyclopedia of Genes and Genomes (KEGG), molecular docking, and virus-host-drug interactome mapping. Chloroquine (CQ) and hydroxychloroquine (HCQ) were utilized as probes to explore the interaction network that is linked to SARS-CoV-2. 47 drug targets were shown to be overlapped with SARS-CoV-2 network and were enriched in TLR signaling pathway. Molecular docking analysis and molecular dynamics simulation determined the direct binding affinity of TLR9 to CQ and HCQ. Furthermore, we established SARS-CoV-2-human-drug protein interaction map and identified the axis of TLR9-ERC1-Nsp13 and TLR9-RIPK1-Nsp12. Therefore, the elucidation of the interactions of SARS-CoV-2 with TLR9 axis will not only provide pivotal insights into SARS-CoV-2 infection and pathogenesis but also improve the treatment against COVID-19.

## Introduction

The outbreak of COVID-19 pandemic caused by SARS-CoV-2 has posed a tremendous threat to mankind (Das et al., 2021a). Viruses require host cellular factors for successful replication during infection. Systematic identification of virus–host protein–protein interactions (PPIs) offers an effective way toward elucidating the mechanisms of viral infection. Subsequently, targeting cellular antiviral targets, such as virus–host interactome, may offer a novel strategy for the development of effective treatments for viral infections (Choudhury et al., 2021a). However, the interaction between the viral antigens and human Toll-like receptors (TLRs) as well as the mechanistic insights of cytokine storm affecting multiple human organs are particularly unknown (Choudhury et al., 2021b).

The activation of the human innate immune cells through the binding of pathogen-associated molecular pattern (PAMP) from SARS-CoV-2 to cell surface TLRs have been demonstrated to be a vital mediator of COVID-19 immunopathogenesis. Being the crucial innate immune sensors and critical mediators of human immunity, manipulation of the activity of TLRs in patients with COVID-19 is currently under consideration for developing effective therapeutics (Catanzaro et al., 2020). Considering the immense importance in the disease biology, TLRs (host)-SARS-CoV-2 interaction appears to be a suitable target for the conception of appropriate therapeutic strategy against the pandemic (Patra et al., 2021).

In the setting of COVID-19, multiple TLRs are likely relevant in viral combat and investigations of TLRs as therapeutic target are starting to emerge (Bezemer and Garssen, 2021). TLR3 recognizes double-strand RNA (ds RNA), TLR4 recognizes lipopolysaccharide (LPS), TLR7/8 recognizes single-strand RNA (ssRNA), and TLR9 recognizes unmethylated CpG DNA (Khanmohammadi and Rezaei, 2021). In contrast to the numerous potential valuable TLR agonists, TLR9 could be considered as particular interesting target of inhibition because of the lack of CpG suppression in unique to SARS-CoV-2 regions which could be of specific concern in vulnerable patients. TLR9 inhibition could thus be a strategy for treatment of the COVID-19 patients that are at risk for developing severe symptomatic infection (Bezemer and Garssen, 2021).

Via the TLR9 pathways, a plethora of inflammatory mediators and cell types can be triggered such as interleukin-6 (IL-6) and tumor necrosis factor (TNF)-α in severely ill patients with COVID-19 (Bezemer and Garssen, 2021; Rubin et al., 2021). The elucidation of the interactions of SARS-CoV-2 with TLR provides pivotal insights into SARS-CoV-2 infection and pathogenesis but also benefit the drug development against COVID-19 (Min et al., 2021). Most of the TLR antagonists can competitively inhibit the binding of spike protein/other viral PAMP to TLR and dampens the expression of IL-6 and TNF- α (Patra et al., 2021).

Recently several repurposed antiviral drugs for COVID-19, including lopinavir/ritonavir (Horby et al., 2020), chloroquine (CQ) and hydroxychloroquine (HCQ) (Ghazy et al., 2020), dexamethasone (Matthay and Thompson, 2020), remdesivir (Young et al., 2021) and molnupiravir have been evaluated for the treatment of COVID-19. Compound-gene network, pathway enrichment analysis, network-based analysis has been separately established to generate key insights on the mechanisms underlying human diseases (Jin and Wong, 2014; Cava et al., 2020; Zhou et al., 2020). Compared with the “one drug, one gene, one pathway” mode, network-based analysis focuses on the “multi-compounds, multi genes, multi pathways” in the treatment of disease and helps to investigate the complex relationships between compounds and targets (Li et al., 2014; Xiong et al., 2018).

**FIGURE 1:**
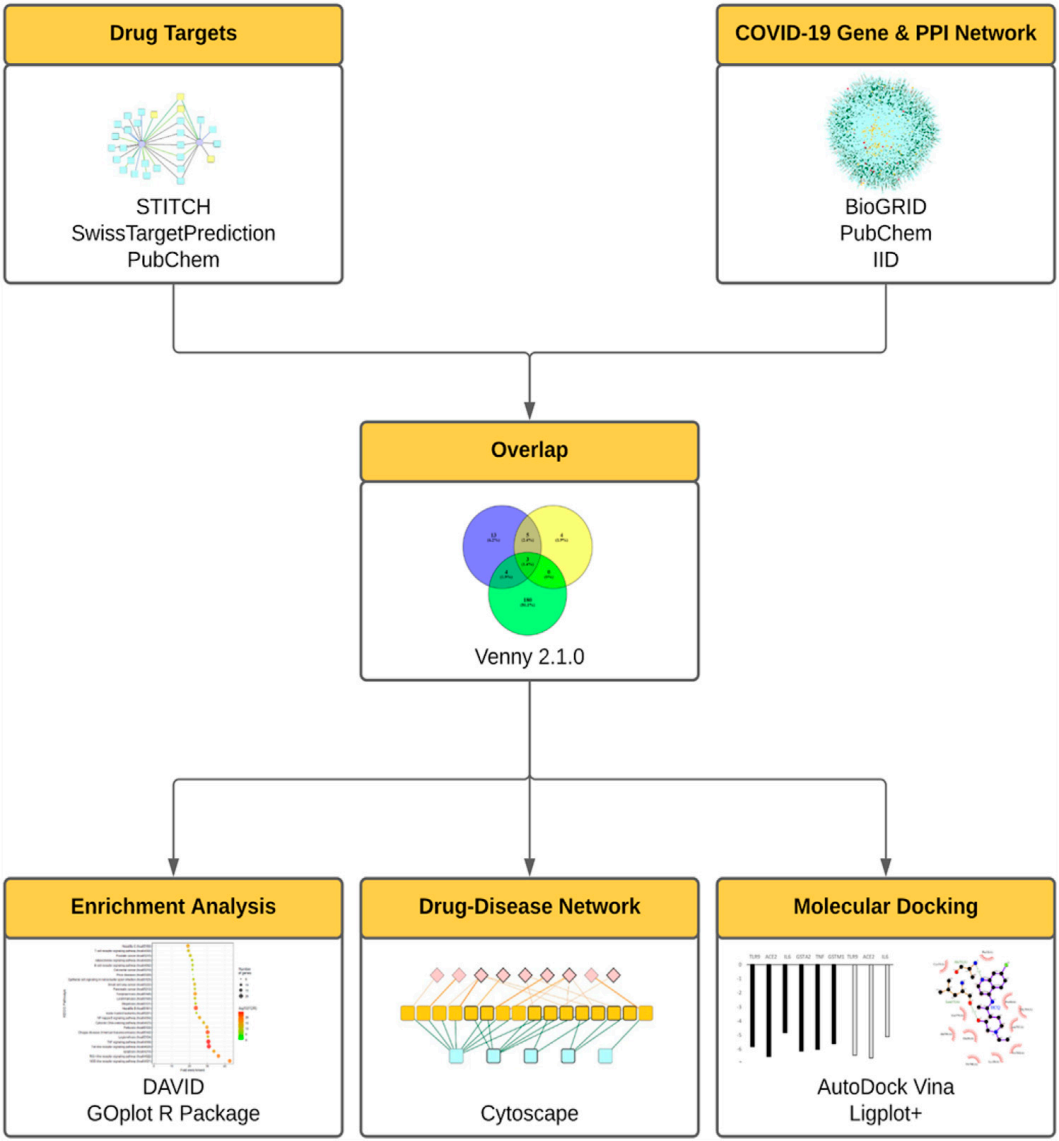
The integrative framework for the assessment of repurposed drugs for COVID-19 treatment. In this framework, drug targets were assessed using STITCH, SwissTarget, PubChem. COVID-19 gene and protein networks were analyzed using PubChem, BioGRID, IID. Then the derived drug targets and gene/protein networks were overlapped (using Venny 2.1.0), then subject to enrichment analysis (DAVID, GOplot R Package), drug-disease network (Cytoscape), molecular docking analyses (AutoDock Vina).

To further integrate the biological and clinical discoveries into the elucidation of the mechanism of repurposed drugs, in this study we established the integrative network medicine framework that incorporated multiple information layers including target identification, compound-gene set enrichment analysis, *in silico* docking study, and virus-host-drug interactome mapping analysis (Figure 1).

## Results

### The Characterization of Drug-Gene Interaction Network Between CQ, HCQ and Gene Targets

**FIGURE 2:**
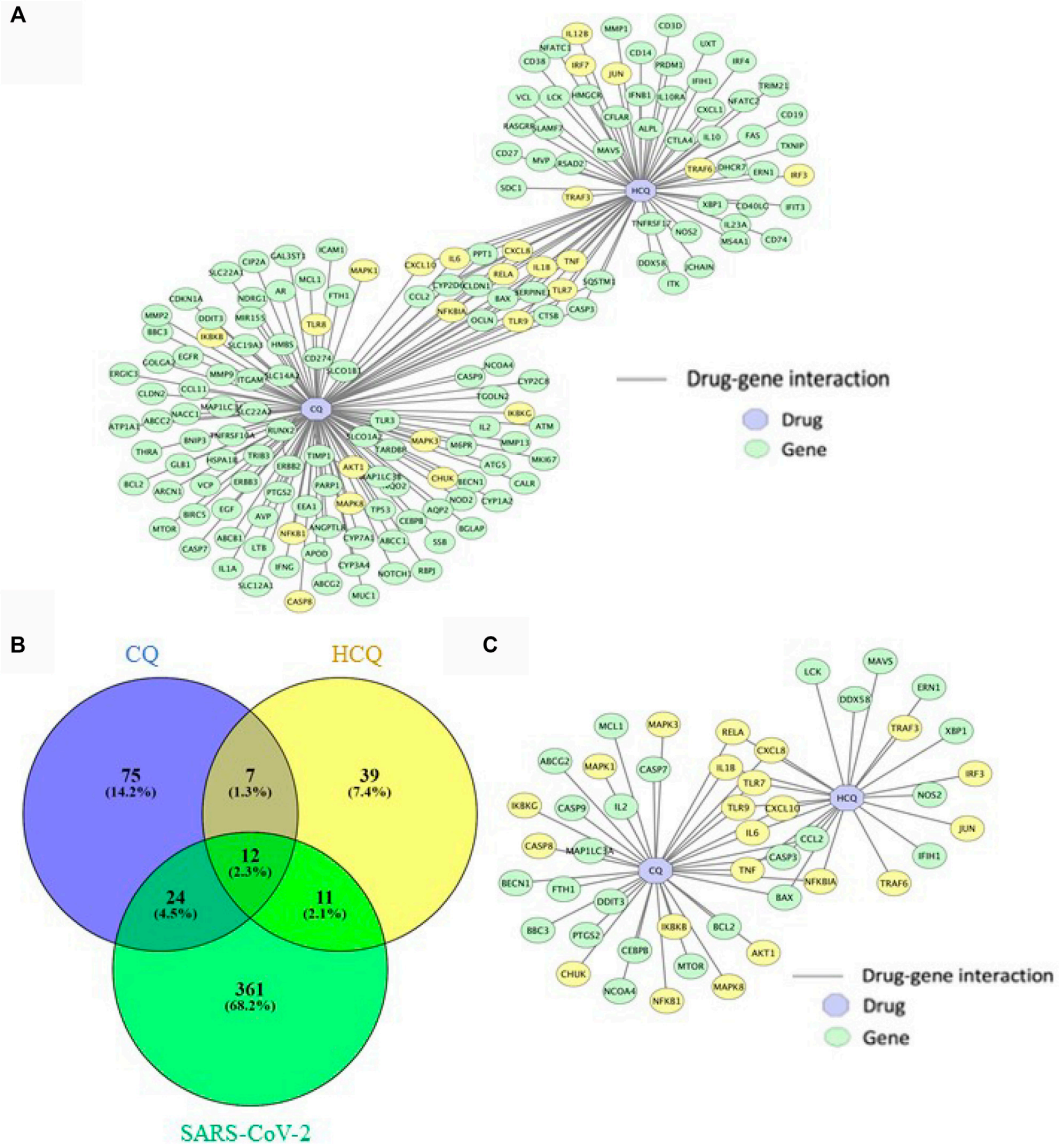
The characterization of drug-gene interaction network between CQ, HCQ, and gene targets. **(A)** Gene interaction network between CQ and HCQ was constructed on Cytoscape. Blue node: drug. Green node: gene. Yellow node: gene in enriched pathways (TLR9 and IL-6 signaling pathways). **(B)** A Venn Diagram visualizing overlap between the gene targets of CQ (blue), HCQ (yellow), and SARS-CoV-2 network (green). 47 overlapped gene targets were identified as CQ/HCQ gene targets of SARS-CoV-2. **(C)** The 47 overlapped gene targets were incorporated to construct a drug-gene interaction network on Cytoscape. Blue node: drug. Green node: gene. Yellow node: gene in enriched pathways (TLR9 and IL-6 signaling pathways).

Utilizing the abundant data from biological and clinical studies on CQ and HCQ (Gasmi et al., 2021), we sought to characterize the drug-gene interaction network and explore the entire mechanisms of CQ and HCQ treatment (including targeted and off-target mechanisms). The overlapping genes between CQ, HCQ and SARS-CoV-2 were identified by a combination of PubChem/SwissTarget/STITICH and constructed using Cytoscape (Figure 2). As displayed in Figure 2, CQ/HCQ-SARS-CoV-2 target network involved 170 nodes (47 common targets and 2 corresponding chemical components) and 189 edges. The results indicated that the 47 nodes might act as potential gene targets for the treatment of COVID-19 using CQ or HCQ. A Venn Diagram visualizing overlap between the gene targets of CQ, HCQ, and SARS-CoV-2 genes identified from PubChem. 47 overlapped target genes were identified (Figure 2B) and then were used to construct a drug-gene interaction network on Cytoscape (Figure 2C). 22 out of 47 genes in the drug-gene interaction network were involved in TLR and IL-6 signaling pathways (highlighted in yellow, Figure 2C). Notably these 22 genes contributed to the common genes (9 out of 12 genes) between CQ and HCQ gene networks (Figure 2C).

### The Gene Targets of CQ and HCQ Were Enriched in TLR and IL-6 Pathways

**FIGURE 3:**
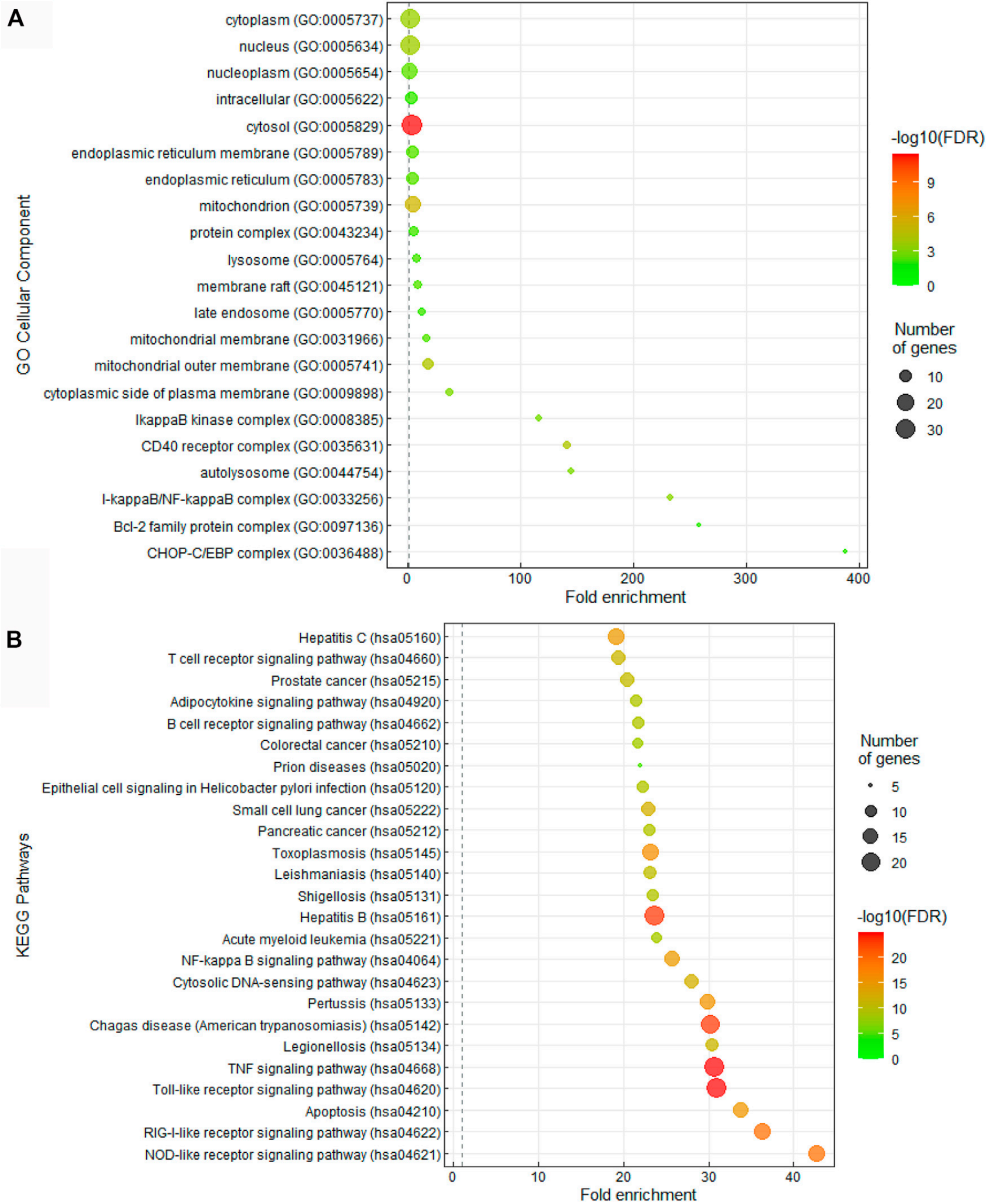
CQ and HCQ targeted TLR and IL-6 signaling pathways. **(A)** Bubble graph representing significant Cellular Components from the 47 overlapped target genes. The size of the dots represents the number of genes that correspond to the enriched GO term. The colorimetry of the dots represents the false discovery rate. **(B)** Bubble graph representing significant KEGG pathways from the 47 overlapped drug target genes from the PubChem database. The size of the dots represents the number of genes that correspond to the enriched KEGG pathway.

Given the interesting correlation of common genes in CQ and HCQ network to TLR or IL-6 signaling pathway (Figure 1), we sought to further elucidate the mechanism of SARS-CoV-2 drug treatment at the system biology level. Thus, we performed Gene Ontology (GO) and Kyoto Encyclopedia of Genes and Genomes (KEGG) enrichment analysis of cellular components and pathways using Annotation, Visualization and Integrated Discovery (DAVID) on the 47 target genes in COVID-19 and CQ/HCQ drug-gene interaction network in Figure 3. The top 25 terms ranked by fold-enrichment and *p* < 0.05 for each analysis were represented using a bubble graph where the most enriched annotations were at the bottom and the number of genes involved were represented by the size of the circles (Figure 3). For cellular components, the functional annotations were associated with Cytosol (GO: 0005829) (Figure 3A). For pathway analysis, 47 gene targets in the COVID-19 and CQ/HCQ drug-gene network were mostly enriched in TLR signaling pathway (GO: 0002224) and nuclear factor kappa light chain enhancer of activated B cells (NF-κB) signaling pathway (GO: 0038061) (Figure 3B).

### Proteins in TLR and IL-6 Pathway Were Shown to Be Direct Targets of CQ and HCQ

**FIGURE 4:**
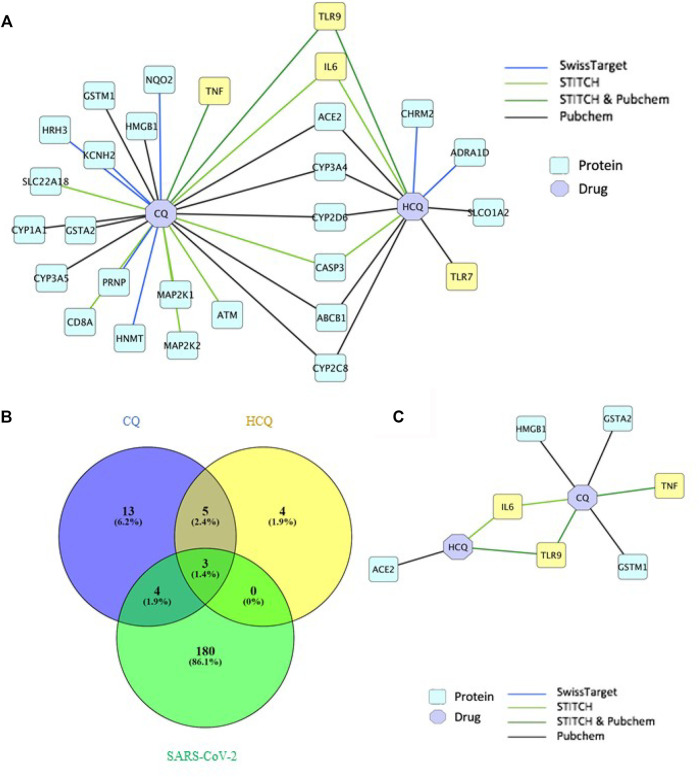
TLR9 and IL-6 were shown to be the direct targets of CQ and HCQ. **(A)** PPI network of interactions between CQ, HCQ and protein targets from 4 databases (SwissTarget, STITCH, STITCH & PubChem, PubChem) was constructed on Cytoscape. **(B)** A Venn diagram was constructed to identify the common protein targets between CQ, HCQ and SARS-CoV-2. **(C)** A drug-protein interaction network was constructed to analyze the 7 common protein targets (Figure 4B) among CQ, HCQ and SARS-CoV-2 using Cytoscape.

The drug-gene network characterization in Figure 2 and GO/pathway enrichment analysis in Figure 3 identified an interesting lead on the mechanism of CQ/HCQ in involving the gene targets from TLR signaling pathway and NF-κB signaling pathway. The characterization of the protein network would deepen the understanding on the mechanism targeting SARS-CoV-2. Therefore, we characterized the protein targets of CQ and HCQ that involved in SARS-CoV-2 network in Figure 4. A protein-protein interaction (PPI) network was constructed using a combination of SwissTarget (Gfeller et al., 2014), STITCH (Kuhn et al., 2008) and PubChem on Cytoscape (Smoot et al., 2011) to identify the direct and indirect drug targets of CQ and HCQ (Figure 4A). Following the overlap with SARS-CoV-2 proteins identified from PubChem, we identified 7 common protein targets (TLR9, IL-6, TNF, ACE2, GSTA2, HMGB1, GSTM1) of CQ and HCQ. Among these targets, four of them (TNF, GSTA2, HMGB1, GSTM1) were shown to be specific to CQ and three of them were the common targets (TLR9, IL-6, ACE2) of CQ and HCQ for SARS-CoV-2 (Figure 4B). These overlapped target proteins were then used to construct a drug-protein interaction network on Cytoscape (Figure 4B).

### TLR9 and IL-6 Were Shown to Have Strong Binding Affinity to CQ and HCQ

**FIGURE 5:**
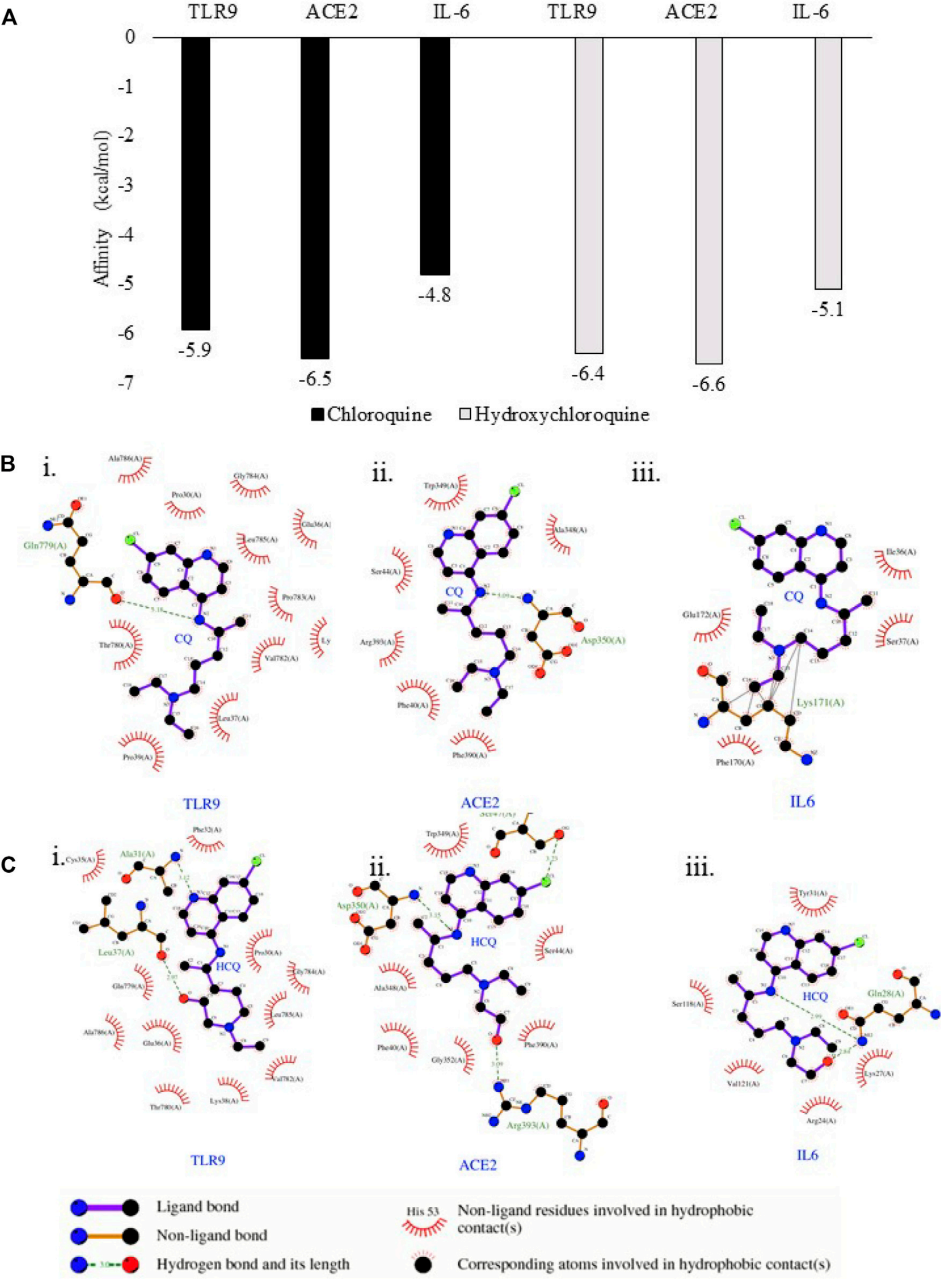
TLR9 and IL-6 exhibited binding affinity to CQ and HCQ. **(A)** Predicted free energy of binding of CQ and HCQ with TLR9, IL-6 and ACE2 were shown by Autodock Vina. **(B)** The output of molecular docking showing the binding site residues of protein targets (TLR9, ACE2 or IL-6) with the ligand CQ. **(C)** The output of molecular docking showing the binding site residues of protein targets (TLR9, ACE2 or IL-6) with the ligand HCQ. The dashed lines represent intermolecular interactions.

Thus, the results in Figure 4 suggested TLR9 and IL-6 were the common protein targets of CQ and HCQ in SARS-CoV-2 network. The three common protein targets (TLR9, IL-6, ACE2) were of significance in the network and thus used for the following molecular docking study in Figure 5. In order to verify the direct interactions between three protein targets (TLR9, IL-6, ACE2) in SARS-CoV-2 network and CQ or HCQ, we performed molecular docking analysis (Figure 5). The docking affinity values between the targets and CQ/HCQ were reported by AutoDock Vina. Meanwhile, the hydrophobic, electrostatic, cation-pi interactions and hydrogen bonds formed in the receptor-spike complex were analyzed using Ligplot+. There were six pairs (TLR9-CQ, ACE2-CQ, IL-6-CQ, TLR9-HCQ, ACE2-HCQ, IL-6-HCQ) delivered into the docking simulation, among which ACE2 was included as positive control due to its direct interaction with CQ and HCQ (Badraoui et al., 2021). The greater the absolute value of the docking affinity, the stronger binding ability between the compounds and the active site of the targets.

The binding affinity of positive control pairs ACE2-CQ docking (−6.5 kcal/mol) and ACE2-HCQ docking (−6.6 kcal/mol) validated this molecular docking system (Figure 5A). The results suggested TLR9 possessed strong binding affinity to CQ and HCQ based upon the analysis of TLR9-CQ docking (−5.9 kcal/mol) and TLR9-HCQ docking (−6.4 kcal/mol). In comparison, IL-6 exhibited weaker binding affinity to both the drugs based on the results of IL-6-CQ docking (−4.8 kcal/mol), IL-6-HCQ docking (−5.1 kcal/mol) (Figure 5A). The detailed interaction patterns were demonstrated in Figure 5B. Among all the pairs of ACE2, TLR9 and IL-6, CQ and HCQ fit into the interface pocket formed by interaction amino acid residues in all three proteins (Figure 5B). The results showed hydrogen bond formation involved in the bonding between CQ and residues in Gln779(A) at TLR9 and Asp350(A) at ACE2. These hydrogen bonds between CQ and TLR9/IL-6 were considered as strong interactions considering the distance of 3.18 Å (TLR9-CQ), 3.09 Å (ACE2-CQ) respectively. In comparison, the hydrogen bond formation between HCQ and residues involved in Leu37(A) at TLR9 and Arg393(A) at ACE2, and Gln28(A) at IL-6. The hydrogen bonds between HCQ and TLR9/IL-6 were also considered as strong interactions given the distance of 2.97 Å (TLR9-HCQ), 3.09 Å (ACE2-HCQ), 2.99 Å (IL-6-HCQ) (Figure 5C). The other essential residues interacted with CQ and HCQ through hydrophobic effects, van der Waals forces, etc. (Figure 5C).

**FIGURE 6:**
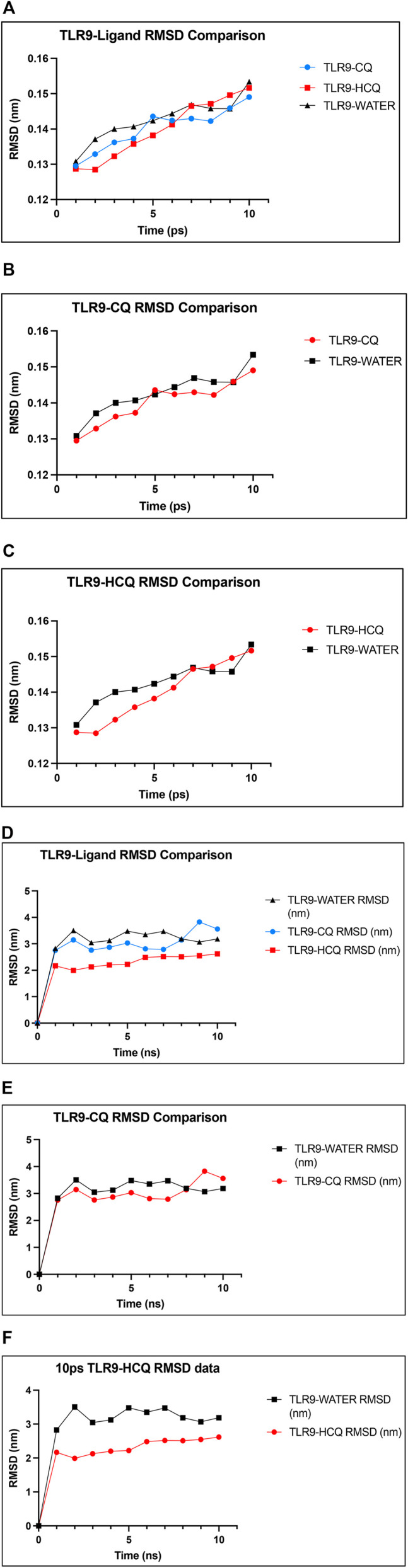
The confirmation of the binding affinity of TLR9 to CQ and HCQ. **(A)** RMSD plot of the TLR9-CQ (shown in blue), TLR9-HCQ (shown in red), TLR9 solvated in water (shown in black) as a function of simulation time. **(B)** RMSD plot of TLR9-CQ and TLR9 solvated in water shows minimal difference between the curves. **(C)** RMSD plot of TLR9-HCQ and TLR9 solvated in water shows minimal difference between the curves. **(D)** A 10 ns time simulation quantifying RMSD of TLR9-CQ (shown in blue), TLR9-HCQ (shown in red), TLR9 solvated in water (shown in black). **(E)** A 10 ns simulation quantifying RMSD of TLR9-CQ vs. TLR9 solvated in water shows minimal difference between the curves. **(F)** A 10 ns simulation quantifying RMSD plot of TLR9-HCQ and TLR9 solvated in water shows significant difference between the curves (*p* < 0.05).

In analyzing the stability of TLR9-ligand complexes we utilized RMSD as the main readout. A larger RMSD value indicates lower stability of the protein complex in the given simulation conditions (Das et al., 2021a; Das et al., 2021b). Comparing RMSD of TLR9 backbone solvated in water and the TLR9-CQ complex it is evident there is minimal differences between the stability of the complex compared to the protein itself with average RMSD values of 0.1402 and 0.1427 nm respectively (*p* > 0.05). Similarly, the average RMSD of TLR9-HCQ was 0.1400 nm showing similar stability to the CQ bound counterpart yet no significant difference exists in average RMSD compared to free TLR9 (Figure 6). In order to further validate any potential differences seen between free TLR9 and the respective ligands we conducted a longer MD simulation to resolve any differences in stability between the complexes. Upon performing a 10 ns simulation the RMSD values stabilized at distinct values. Average RMSD values of 3.226, 3.069, and 2.339 nm were observed for TLR9 solvated in water, TLR9-CQ, and TLR9-HCQ respectively (Figure 6). Coinciding with binding affinity the average RMSD value of TLR9-HCQ showed a significant difference (*p* < 0.05) compared to free TLR9 while the same comparison for TLR9-CQ yielded no significance. This suggests the deviation of atoms between TLR9 and HCQ is less than that observed between TLR9 and CQ.

### TLR9 Was Associated With SARS-CoV-2 Proteins Nsp12, Nsp13, ORF8 and E in SARS-CoV-2-Host-Drug Interactome

**FIGURE 7:**
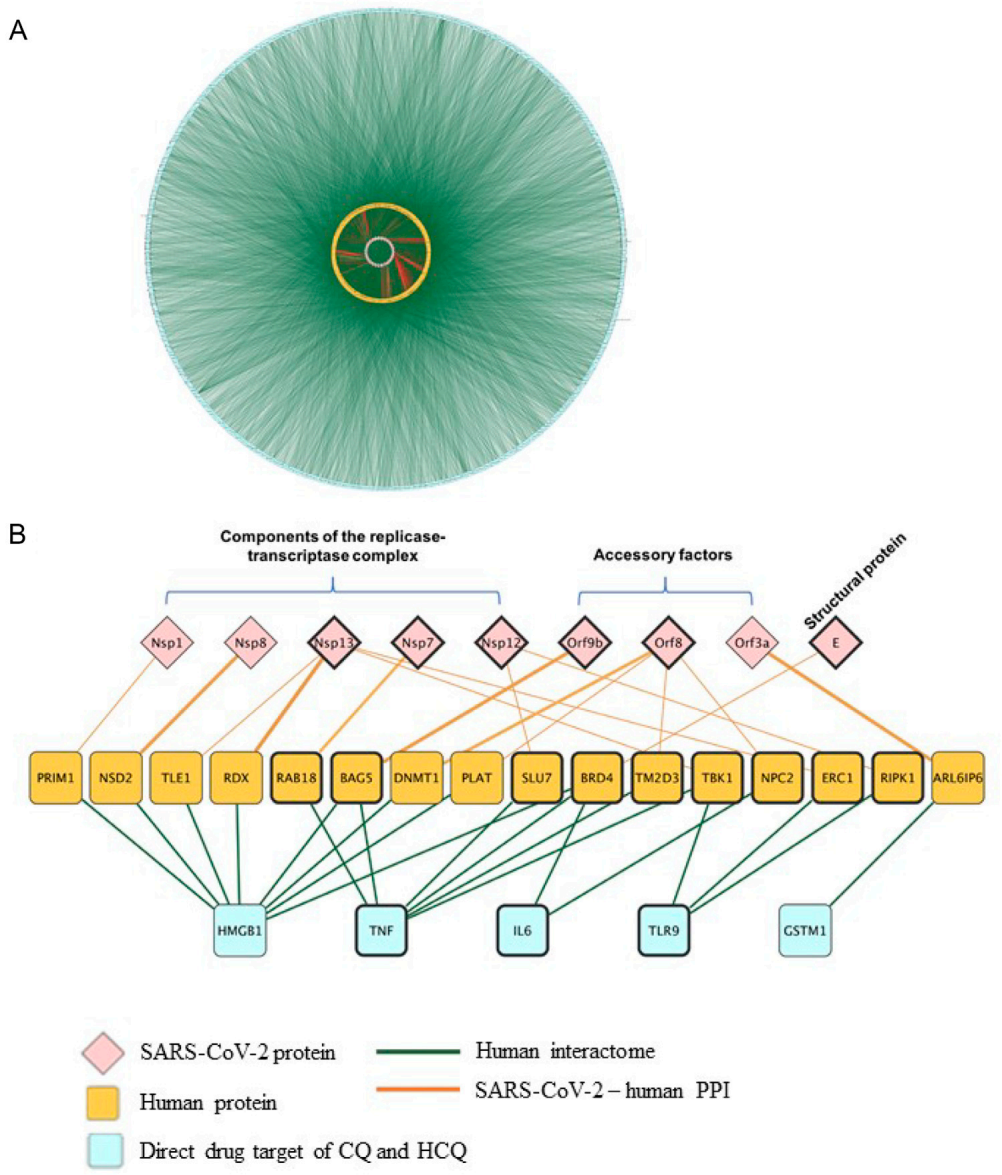
The identification of the interactions between TLR9 and Nsp12/Nsp13 in SARS-CoV-2-host interactome. **(A)** Interactions between SARS-CoV-2 proteins with their direct human protein partners (green line: human interactome; yellow circle: human proteins; red line: SARS-CoV-2-human PPI). **(B)** A close-up view showing the interactions between the CQ and HCQ drug targets and human proteins that were found to interact with SARS-CoV-2 proteins.

Molecular docking results in Figures 5, 6 confirmed TLR9 as direct targets of CQ and HCQ. Then the next question is how we can use the tools of CQ and HCQ to explore the possibility of repurposed drug development to disrupt the SARS-CoV-2 network in human body. To address this question, we have established SARS-CoV-2-human-CQ/HCQ interactome in Figure 7 and investigated the connectivity between TLR9 and IL-6, their PPIs and SARS-CoV-2 proteins that play a critical role in SARS-CoV-2 pathogenesis. By merging direct interaction partners of SARS-CoV-2 proteins from BioGRID with the human-protein interactions from Integrated Interactions Database (IID), we generated the SARS-CoV-2-human-CQ/HCQ interactome map (Figure 7A). As constructed on Cytoscape, the innermost circle were SARS-CoV-2 proteins, followed by the orange layer of direct targets, and the blue outer circle of secondary proteins (Figure 7A).

Using Diffusion, a network propagation algorithm for Cytoscape (Oughtred et al., 2019), we constructed a close-up view showing the interactions between the CQ and HCQ drug targets (TLR9 and IL-6) were associated with human proteins (BRD4, TBK1, NPC2, ERC1, RIPK1) that were found to interact with SARS-CoV-2 proteins components of the replicase-transcriptase complex (Nsp12, Nsp13), accessory factors (ORF8) and structural protein (E) (Figure 7B). Among these interactions, SARS-CoV-2 replicase-transcriptase complex (Nsp12 and Nsp13) was targeted by TLR9. As targeting IL-6 is a potential therapeutic strategy for COVID-19 (Y and R, 2021), our results showed that IL-6 targeted replicase-transcriptase complex (Nsp13), accessory factor (ORF8) and structural protein (E) (Figure 7B). Specifically, the role of TLR9 on SARS-CoV-2 human interactome were achieved through the interaction of TLR9-TBK1-Nsp13; TLR9-ERC1-Nsp13; TLR9-RIPK1-Nsp12 (Figure 7B).

## Discussion

System biology plays a vital role in this global scenario to fight against COVID-19 (Jaiswal et al., 2020). Previously we combined meta-analysis, gene expression omnibus (GEO) data and animal models results to identify ACE2 as an indicator of the susceptibility to SARS-CoV-2 infection (Kong et al., 2020). Although much attention in biomedical and clinical research is given to the task of identifying therapeutically exploitable drugs (Sadegh et al., 2020; White et al., 2020; Zhou et al., 2020), the mechanism of such candidates remains unclear and this prevented the drug development for SARS-CoV-2.

Given the abundance of biological and clinical data associated with CQ and HCQ, we utilized them as tools to identify the drug targets, pathways, and interaction network against SARS-CoV-2. Thus, this framework will also be applicable to other repurposed drugs. From the drug-gene interactions of CQ and HCQ retrieved from four databases (SwissTarget, STITCH, STITCH & PubChem, PubChem), 47 gene targets were identified as the overlap among COVID-19 and CQ/HCQ drug-gene interaction network (Figure 2).

These gene targets in SARS-CoV-2 and CQ/HCQ drug-gene interaction network were further investigated at the system biology level using GO and KEGG enrichment analysis of Cellular Components and KEGG pathways using DAVID. Figure 3 showed that 47 gene targets in SARS-CoV-2 and CQ/HCQ drug-gene network were enriched in TLR signaling pathway and NF-κB signaling pathway. A PPI network was constructed using a combination of SwissTarget, STITCH and PubChem on Cytoscape to identify the direct and indirect drug targets of CQ and HCQ from TLR signaling pathway and NF-κB signaling pathway (Figure 4). Through this protein-drug network, we identified 7 common protein targets (TLR9, IL-6, TNF, ACE2, GSTA2, HMGB1, GSTM1) of CQ and HCQ. Among these targets, four of them (TNF, GSTA2, HMGB1, GSTM1) were unique to CQ and three of them were the common targets (TLR9, IL-6, ACE2) of CQ and HCQ in SARS-CoV-2 (Figure 4B).

CQ and HCQ prevent virus–cell fusion through the direct binding of the SARS-CoV-2 Spike protein to ACE2 (Mauthe et al., 2018; Ghazy et al., 2020). Since both CQ and HCQ were found to interact differently with ACE2 (Badraoui et al., 2021), we used ACE2 as a positive control to validate the molecular docking system and identified TLR9 and IL-6 as direct targets of CQ and HCQ (Figure 5).

Through our virus-host-drug interactome network (Figure 7), we showed that CQ and HCQ direct drug targets (TLR9 and IL-6) were associated with human proteins (RAB18, BAG5, SLU7, BRD4, TM2D3, TBK1, NPC2, ERC1, RIPK1) that were found to interact with SARS-CoV-2 proteins Nsp12, Nsp13 (Components of the replicase-transcriptase complex), ORF8 (Accessory factors), and E (Structural protein) (Figure 7B). Among these interactions, SARS-CoV-2 replicase-transcriptase complex (Nsp12 and Nsp13) was targeted by TLR9. In comparison, IL-6 targeted replicase-transcriptase complex (Nsp13), accessory factor (ORF8) and structural protein (E). As the common SARS-CoV-2 protein that was targeted by TLR9 and IL-6 (Khaedir and Kartika, 2021), Nsp13 is a helicase that unwinds SARS-CoV-2 RNA, which is essential in the viral life cycle (Chen et al., 2020; Shu et al., 2020). Thus, we established SARS-CoV-2-human-drug protein interaction map and identified the axis of TLR9-ERC1-Nsp13 and TLR9-RIPK1-Nsp12. In this context, our study depicts the identity of the intracellular TLR9-binding partners from SARS-CoV-2 and their biophysical interactions with the TLRs. Taken together, our findings are expected to provide a new dimension to the existing knowledge of the immunobiology of TLRs and it will promote future studies for the conception of intervention strategies.

We acknowledge that there are some limitations in this current study. First, owing to a lack of the complete drug-target information, other drugs such as dexamethasone and remdesivir were not included in the study (Supplementary Figure S1). Second, the collected virus–host interactions are far from completeness and the quality can be influenced by multiple factors, including different experimental assays and human cell line models. However, we expect that our methodology remains to be a useful network-based tool for prediction of multiple drugs toward exploring network relationships of multiple drugs’ targets with the SARS-CoV2–host subnetwork in the human interactome. Thus, the findings in the current study may improve coverage of current network-based methodologies by utilizing multi-layer network framework.

In summary, we have presented an integrative framework for the interactive exploration and network-based analysis of virus–host interactions, aimed towards drug repurposing for the treatment of COVID-19. This framework can be easily adapted to accommodate the fast-paced data generation in the battle against the global pandemic. This system is expected to speed up the discovery of potential therapeutics for SARS-CoV-2 variants that are resistant to vaccination.

## Conclusion

This research gives an insight into the applicability of systems biology tools for developing drugs against COVID-19 infection. We identified 47 gene targets as the overlap among CQ/HCQ drug-gene interaction network and SARS-CoV-2 network. These genes were shown to be enriched in TLR signaling pathway, from which we identified TLR9 as the direct drug target of CQ and HCQ. The *in silico* application of molecular interaction and simulation study was done purposely for the understanding of host (human) and virus interaction to plan the future strategies for managing the situations of virus pandemics.

## Methods

### Identification of Protein and Gene Targets

Drug-Protein targets of CQ and HCQ were identified through 3 different databases: The Search Tool for Interactions of Chemicals (STITCH) (http://stitch.embl.de/), SwissTargetPrediction (http://www.swisstargetprediction.ch/), and PubChem (https://pubchem.ncbi.nlm.nih.gov/). On STITCH, the minimum required interaction score was set to high (0.700) and the search was conducted within the *Homo sapiens* organism. On SwissTargetPrediction, the minimum probability of interaction was set at 0.50. Gene targets of hydroxychloroquine and chloroquine were identified through the PubChem database under “Chemical-Gene Interactions.” Proteins and genes involved in SARS-CoV-2 infection were identified under the PubChem database.

### Network Construction

The network visualization tool Cytoscape 3.6.1 (http://cytoscape.org/, ver. 3.5.1) was adopted to obtain the PPI network map. Common targets between the compound-putative target network and SARS-CoV-2 target PPI network were identified as potential targets for the components of CQ and HCQ in COVID-19. In such a network, a compound or a gene/protein serves as an “edge” and reflects an association between nodes.

### GO and Pathway Enrichment Analysis

In order to clarify the functions of the potential targets and their involvement in signaling pathways, GO enrichment and KEGG pathway enrichment analyses of the targets in the compound-COVID-19 target network were performed using the Database for DAVID 6.8 (https://david.ncifcrf.gov/). The GO project elucidates the biological significance of a set of genes through three categories: cellular component, molecular function, and biological process. The 47 overlapping genes from the COVID-19-compound network were inputted into the DAVID database to reveal gene functions that were overrepresented among the set. KEGG pathway analysis which reveals pathway functional annotations of a gene set, was also performed using the 47 overlapping genes through the DAVID platform. The top 25 terms with the highest fold enrichment values and *p*-value < 0.05 from the GO and KEGG analyses were chosen and visualized using the “GOplot” package in R software.

### Molecular Docking

The crystal structures of TLR9 (3WPF, 1.96 Å), ACE2 (1R42, 2.20 Å), IL-6 (4O9H, 2.42 Å) were retrieved from the Protein Data Bank (https://www.rcsb.org). Using AutoDock Tools 1.5.6, the complexes were prepared by removing the original ligand, water molecules, and other structures, leaving only the protein of interest. Next, polar hydrogen bonds were added, and the structures were saved in .pdbtq format.

The 3-D structures of the ligands CQ (CID_2719) and HCQ (CID_3652) were obtained from PubChem (https://pubchem.ncbi.nlm.nih.gov/) in .sdf format. The molecular geometries of the ligands were then optimized using Avogadro 1.2.0 (https://avogadro.cc/) with Force Field type MMFF94 and saved in .pdb format.

All ligand and receptor files were saved as. pdbqt format. Then we used Autodock Vina, a freely available open-source package, to evaluate and verify the binding affinity of compound-target relationship, and the prediction results from network pharmacology. A grid box was generated to cover the entire protein with the X, Y, Z coordinates set at the center of the macromolecule. Furthermore, the exhaustiveness was set at 24 as per the published protocol by Forli et al. The binding models were visualized by PyMol2.3.0 software and Ligplot+ 2.2. software.

### Molecular Dynamic Simulation

The docked output structures from molecular docking of TLR9 and ACE2 were converted to pdb files through PyRx (https://pyrx.sourceforge.io/) & BIOVIA Discovery Studio Visualizer (https://discover.3ds.com/discovery-studio-visualizer-download) using the confirmation of either CQ or HCQ that had the lowest vina result value (Model 1) along with the protein without the ligand serving as controls. Charmm-gui’s solution builder (https://www.charmm-gui.org/?doc=input/solution) was then used to create the cubical water box, fitted to the protein complex, the force field, and protonation states. Charmm-gui was also used to add counter ions to make the system electrically neutral before each molecular dynamic simulation. NAMD software (https://www.ks.uiuc.edu/Research/namd/) was then used to run the equilibration step using canonical NVT and NPT ensembles at a stable temperature of 300 K and pressure of 1 bar. The production step followed using canonical parameters optimized through Charmm-gui. Once all files were made, VMD software (https://www.ks.uiuc.edu/Research/vmd/) was used to visualize the molecule and run the simulation by loading the input file of the protein or complex and loading the production dcd file into it. With VMD’s RMSD trajectory tool, RMSD graphs were produced with RMSD (nm) on the *y*-axis and time on the *x*-axis.

### Retrieval and Analysis of the Human-SARS-CoV-2 Protein-Protein Network

Human proteins that directly interact with SARS-CoV-2 proteins identified by Gordon et al. were retrieved from the BioGRID database (https://thebiogrid.org/220839/publication/a-sars-cov-2-protein-interaction-map-reveals-targets-for-drug-repurposing.html). In order to explore the relationship of these protein with the human PPI interactome, we expanded the network by inputting the human proteins into the Integrated Interactions Database (IID) (http://iid.ophid.utoronto.ca/), which is an extensive interaction network that includes PPIs from 9 curated databases, PPIs from orthologues, and high confidence PPIs from established computational methods. We then mapped the output PPI from IID onto Cytoscape 3.6.1 (https://cytoscape.org/, ver. 3.5.1) and merged it with the SARS-CoV-2 human PPIs. Using Cytoscape’s built-in diffusion algorithm, we traced the interaction of the proteins targeted by CQ and HCQ to the SARS-CoV-2 viral proteins.

## Data Availability

The original contributions presented in the study are included in the article/Supplementary Material, further inquiries can be directed to the corresponding author.
